# The usefulness of virtual, augmented, and mixed reality technologies in the diagnosis and treatment of attention deficit hyperactivity disorder in children: an overview of relevant studies

**DOI:** 10.1186/s12888-021-03632-1

**Published:** 2022-01-04

**Authors:** Saeideh Goharinejad, Samira Goharinejad, Sadrieh Hajesmaeel-Gohari, Kambiz Bahaadinbeigy

**Affiliations:** 1 Medical Informatics Research Center, Institute for Futures Studies in Health, Kerman University of Medical Sciences, Kerman, Iran; 2 Department of Health Information Management, School of Allied Medical Sciences, Tehran University of Medical Sciences, Tehran, Iran

**Keywords:** Virtual reality, Augmented reality, Mixed reality, Attention deficit hyperactivity disorder

## Abstract

**Background:**

Attention deficit hyperactivity disorder (ADHD) is a neurodevelopmental condition characterized by attention problems, excessive physical activity, and impulsivity. ADHD affects not only the patients but also their families. The development and use of technologies such as virtual reality (VR), augmented reality (AR), and mixed reality (MR) for ADHD has increased over recent years. However, little is known about their potential usefulness. This overview aimed to clarify the current knowledge about the use of these three innovative technologies for the diagnosis and treatment of children with ADHD.

**Methods:**

This overview was conducted using the PubMed, Web of Science, and Scopus databases until January 24th, 2021. The following descriptive information was compiled from the identified studies: country, year of publication, sample size, study design, ADHD diagnosis methods, applied technology, hardware equipment, clinical target, and main findings.

**Results:**

The initial database searches yielded 409 articles, but 103 were removed as duplicates. Eventually, 30 eligible studies remained for analysis, the majority of which were case-control (*n* = 22, 73%). Regarding the applied technology/hardware equipment, VR (*n* = 27; 90%), head-mounted displays (*n* = 19, 63%), VR-based continuous performance tests (VR-CPT) (*n* = 21, 70%) were most frequently used. Most studies (*n* = 21, 70%) used the DSM criteria for the diagnosis of childhood ADHD. They primarily evaluated the utility of these technologies in assessing ADHD symptoms (*n* = 10, 33%) and improving the ADHD diagnostic process (*n* = 7, 23%).

**Conclusion:**

This comprehensive overview evaluated the studies on the use of VR, AR, and MR technologies for children with ADHD. These technologies seem to be promising tools for improving the diagnosis and management of ADHD in this population.

**Supplementary Information:**

The online version contains supplementary material available at 10.1186/s12888-021-03632-1.

## Introduction

Attention deficit hyperactivity disorder (ADHD) is a prevalent mental disorder in children and adults [1] with its prevalence rate varying among different age groups [2]. ADHD affects approximately 4–12% of school-aged children worldwide [3, 4]. It is a syndrome characterized by a short attention span, impulsivity, and hyperactivity that often lead to multiple behavioral problems [5]. ADHD is classified into three categories of combined ADHD (highest prevalence), impulsive/hyperactive ADHD, and inattentive/distractible ADHD [6].

ADHD causes primary and secondary complications such as a lack of self-confidence, relationship maladjustment with friends, and incompatibility with social and academic environments [7, 8]. It is also associated with heavy healthcare costs since it increases the risk of major disorders such as depression, bipolar disorder, and anxiety in the patients [9]. ADHD can also contribute to hypertension, obesity, diabetes, asthma, migraine, epilepsy, and dyslipidemias [10]. Therefore, it is essential to properly diagnose the symptoms of this disorder and take effective measures to ameliorate the main symptoms and other clinical comorbidities [11].

Medication and cognitive-behavioral therapy (CBT) [12] are the primary treatment options for ADHD. Psychopharmacological treatment such as the prescription of methylphenidate is not always effective and may have serious side effects [13]. Low medication adherence is another common problem among children diagnosed with ADHD [14]. CBT involves targeted cognitive and behavioral therapeutic measures used for the treatment of multiple psychosocial disorders. In effective CBT, patients must have several sessions with a therapist who specializes in ADHD [15]. Other treatment options such as memory, speech, and family therapy are also effective in diminishing the adverse impact of ADHD symptoms on children [16, 17]. Unfortunately, these primary treatment options have potential limitations, such as medication side effects, lack of behavioral improvement, high costs, and major time commitments [18, 19]. In the treatment of children with ADHD, it is essential for the children to learn self-control and how to make and keep friends while developing a good sense of self-esteem [20].

Recently, technological advancement has enabled the use of mobile phone applications, telemedicine, computer/mobile games, continuous performance tests (CPT), virtual reality (VR), and augmented classroom simulators for ADHD diagnosis and treatment [21–23]. VR is a state-of-the-art, technologically advanced system that simulates three-dimensional (3D) environments in which an individual can become fully immersed and have a realistic experience [24–26].

Augmented reality (AR) is a subset of VR consisting of real-world features, digital information, and elements that enable users to interact with virtual objects and view the physical environment [27, 28]. VR transmits information from the physical environment to an entirely virtual world, whereas AR merges virtual objects into a real-world environment [29]. Consequently, VR allows users to feel psychologically immersed in a virtual environment, while AR provides an environment to let users interact with virtual objects in the real world [30]. Mixed reality (MR) falls somewhere between AR and VR as it is a mixture of actual and virtual reality whereby the user can observe the real world as in AR and observe realistic virtual objects as in VR [31, 32]. In other words, MR allows the users to interact with virtual elements within their real-world experience [33].

Several studies have indicated that VR and AR tools are remarkably effective in the promotion of general health, mental health treatment, and diagnosis [34, 35]. VR, AR, and MR have been integrated into the treatment of various mental disorders. For instance, a study by Smith et al. [36] demonstrated that VR could be used for the rehabilitation of patients with schizophrenia. Mclay et al. [37] reported the application of VR for the management of posttraumatic stress disorder (PTSD). Lee et al. [38] and Magrini et al. [39] also employed AR and Liu et al. [40] used MR to improve the symptoms of children with the autism spectrum disorder.

These technologies provide a virtual environment that allows individuals to experience various situations that may be difficult or even impossible to deal with in reality; as such, they are more effective and safer than traditional treatments [41]. In these environments, users can develop different skills and a greater understanding of their problems, which helps them to better control their behavior in similar real-world situations [42, 43].

VR, AR, and MR technologies are particularly effective with the design of attractive virtual environments that engage and increase users’ attention. Several studies have explored the use and effectiveness of VR, AR, and MR technologies in the treatment and diagnosis of children with ADHD [44–46]. This study aimed to compile and describe these studies.

## Materials and methods

First, we searched for relevant articles on ADHD published until January 24th, 2021, and available on PubMed, Web of Science, and Scopus. The articles were retrieved using various keywords, including ((virtual reality) OR (augmented reality) OR (mixed reality)) AND ((attention deficit hyperactivity disorder) OR (ADHD)). The terms included in the search based on the PICO guideline are presented in Table 1.

**Table 1:** Key Search Terms

PICO	Key Search Term
Population	(attention deficit hyperactivity disorder) OR (ADHD)
Intervention	(virtual reality) OR (augmented reality) OR (mixed reality)
Comparison	Based on the inclusion and exclusion criteria
Outcome	Based on the inclusion and exclusion criteria

Articles were selected in accordance with the PICO guidelines as follows: P-population (children and adolescents aged 4–18 years diagnosed with ADHD), I-intervention (using VR/AR/MR for ADHD diagnosis and management), C-comparison (VR/AR/MR intervention group vs. control group or groups before and after VR/AR/MR intervention), and O-outcome (the main outcomes of VR/AR/MR interventions).

The inclusion criteria were being written in English, with a focus on VR, AR, or MR in ADHD patients, and recruiting patients younger than 18 years. Publications were excluded if they reported no data on VR, AR, and MR outcomes, if they were unavailable in full text, and if they were reviews, abstracts, notes, protocols, letters, or editorials,

### Quality assessment

Two independent reviewers (Saeideh Goharinejad and Samira Goharinejad) assessed the methodological quality of the studies based on the Joanna Briggs Institute (JBI) [47]. The JBI has different checklist items for each study design, i.e., case-control, cross-sectional, case studies, and randomized controlled trials. It also evaluates the extent to which a study addresses the potential biases in different aspects of research (e.g., sample size, study design, study procedure, confounding factors, and data analysis) [48].

## Results

**Fig. 1:**
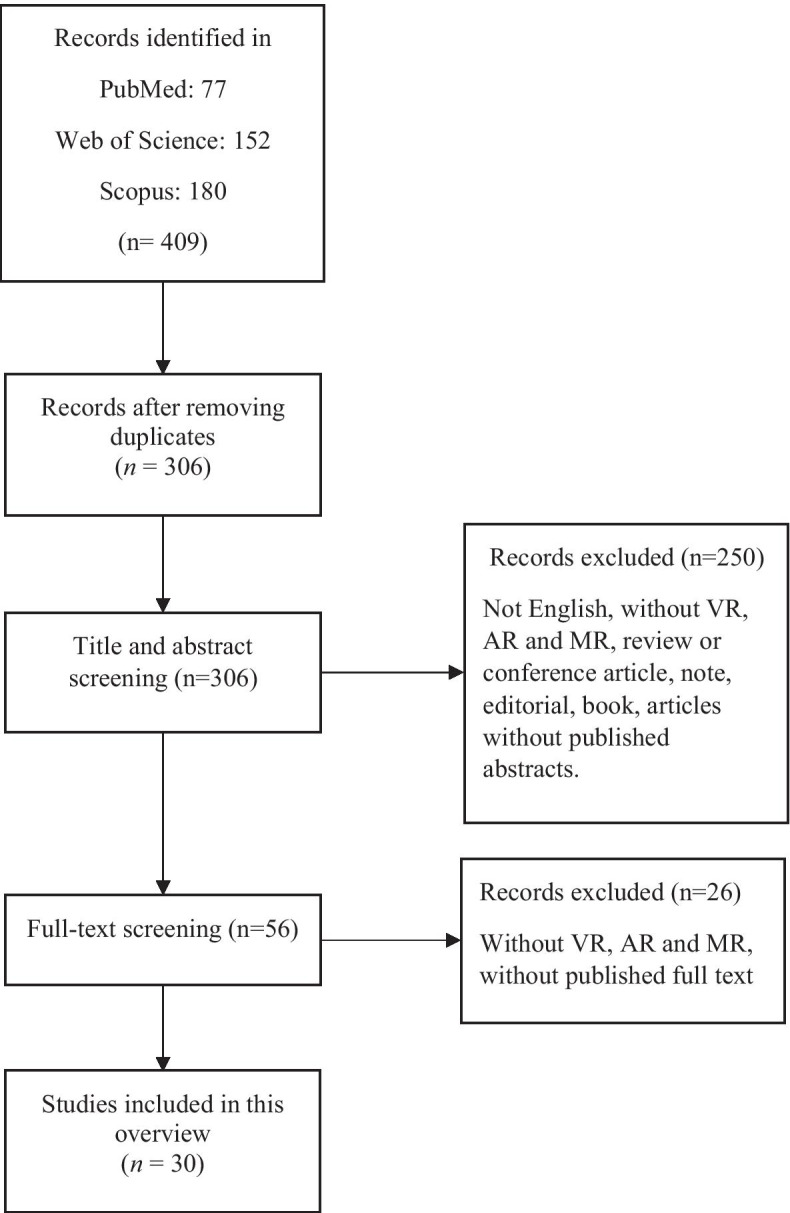
Flow Chart of Data Collection and Analysis

Initially, 409 articles were retrieved from the aforementioned databases. After eliminating 103 duplicates, 306 articles remained, the titles and abstracts of which were screened by two authors independently (Saeideh Goharinejad and Samira Goharinejad). Disagreements were resolved by consulting a third author (Sadrieh Hajsmaeel-Gohari). At this stage, 250 articles were also eliminated as they did not meet the inclusion criteria. Following that, the full texts of the remaining 56 articles were screened by two authors independently (Saeideh Goharinejad and Samira Goharinejad), and 26 articles were eliminated based on the exclusion criteria. Finally, 30 articles were selected for the final review (Fig. 1), and the same two authors independently compiled the descriptive data collected from these articles. This data included the first author’s name, year of publication, country of publication, sample size, study design, ADHD diagnosis methods, clinical target, type of applied hardware technology, type of applied VR, AR, or MR technology, and significant findings.

**Fig. 2:**
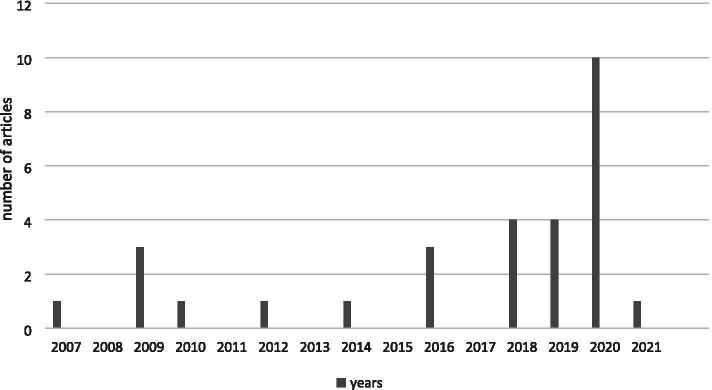
Year of Publication

**Fig. 3:**
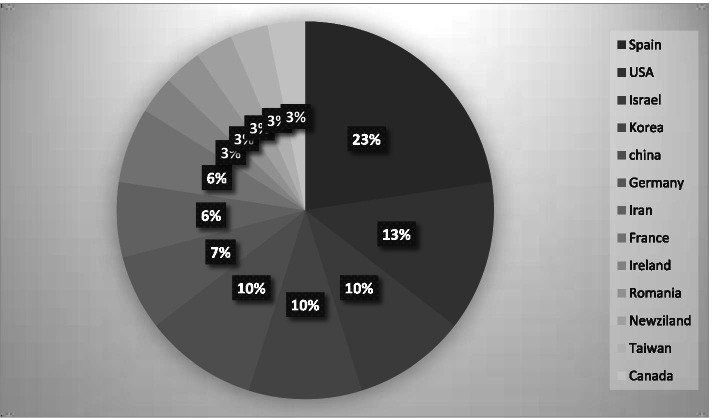
Frequency of Articles by Country

Twenty-five articles were published after 2010 (83%), 10 (33%) in 2020, four in 2018 and 2019 (13%), and three between 2009 and 2016 (10%) (Fig. 2). In terms of country, the most articles were published in Spain (*n* = 7; 23%), the United States (*n* = 4; 13%), and Israel, Korea, and China (all with *n* = 3; 10%) (Fig. 3).

In total, 2378 participants were enrolled in the selected studies with ages ranging from 4 to 18 years. The majority of the participants were male (*n* = 1687; 73%), while two studies did not specify the gender of the participants. The number of the studied patients varied from case studies (*n* = 2; 7%) to larger samples of > 100 patients (*n* = 7; 23%) (Table 2).

**Table 2:** VR, AR, and MR Used for Children with ADHD

Reference	Number of Participants, Age, Sex,	Study Design	ADHD Diagnosis Methods	Clinical Target	Hardware technology, type of technology	Significant Findings
Adams et al. 2009, USA [49].	35 (8–14) y/o, (35 M)	Case, Control Experimental (children with ADHD, *n* = 19 Comparison (Control: *n* = 16)	-licensed mental health professionals or pediatric physicians -parent confirmation	Diagnosis of ADHD	Elumens dome system, head-tracking sensor/VRC_CPT	VRC_CPT has an overall better ADHD classification rate than standard CPT (88% versus 69%).
Areces et al., 2016, Spain [50].	*n* = 117 (5–16) y/o (90 M,27F)	Case, Control Experimental (inattentive ADHD: *n* = 28 I/H ADHD: *n* = 29 ADHD combined: *n* = 32) Comparison (control: 28)	-DSM-5	Diagnosis of ADHD	HMD equipped with motion sensors and headphones/ VRC_CPT	VRC_CPT classified 67% of control and 57% of ADHD combined participants correctly; standard CPT classified 60% of control and 50% of ADHD combined participants correctly.
Areces et al., 2018, Spain [51].	*n* = 88 (6–16) y/o (66 M,22 F)	Case, Control Experimental (children with ADHD: *n* = 50) Comparison (Control *n* = 38)	-DSM-5	Diagnosis of ADHD	3D virtual glasses/VRC_CPT	Omission errors on VCR-CPT had a classification rate of 66% for the control group and 89% for the ADHD group.
Areces et al., 2020, Spain [52].	*n* = 150 (5–16) y/o (114 M, 36 F)	Cross-sectional children with ADHD: *n* = 150	-DSM-5	Assessment of ADHD symptoms	(3D) glasses equipped with motion sensors and headphones/VR_CPT	EDAH ADHD observation inattention subscale predicts VR-CPT omission errors with 86% accuracy, commission errors with 80% accuracy, and response time with 74% accuracy.
Arpaia et, al. 2020, Italy [45].	*n* = 4 (6–8) y/o	Case Study Children with ADHD	Not identified	Concentration	AR/Glasses, Acquisition Unit, Processing Unit, Robot/AR, Robot	Controlling a robot in an AR environment enhanced attentional performance to 83% accuracy.
Bioulac et al., 2012, France [53].	*n* = 36 (7–10) y/o (36 M)	Case, Control Experimental (children with ADHD: *n* = 20) Comparison (Control: *n* = 16)	-DSM-IV -interviews with children and parents -The Conners’ Parent Rating Scale (CPRS)	Assessment of ADHD symptoms	HMD/ VRC_CPT	VRC_CPT is a reliable method to assess the ability to sustain attention over time. VRC-CPT variables correlate with standard CPT (CPT-III) measures.
Bioulac et al. 2018, France [44].	*n* = 51, (7–11) y/o (41 M, 10 F) RCT	Randomized Control Trial Experimental (children with ADHD: *n* = 16) Comparison (methylphenidate group: *n* = 16, psychotherapy group: *n* = 16)	-DSM-IV -interviews with children and parents	Concentration	HMD/ VRC_CPT	The VR cognitive remediation program reduces distractibility.
Blume et al. 2018, Germany [54].	*n* = 81, MA (11.27) y/o, (46 M,35 F)	Cross-sectional The sample covers a wide range from low to high intensity of ADHD symptoms.	-The Conners Rating Scale	Education	HMD/ VRC	The learning outcomes of students with ADHD are not better for those seated close to vs. distant from the teacher.
Cho et al., 2002, China [55].	*n* = 50 (14–18) y/o	Case, Control Experimental (children with ADHD1: *n* = 10, children with ADHD2: *n* = 10) Comparison (placebo groups1 = 10, placebo group2 = 10 Control: *n* = 10)	-Not identified	Concentration	HMD/ VRC_CPT	VR cognitive training improved the patients’ performance on the standard CPT.
Clancy et al., 2016 New Zealand [56].	*n* = 48(13–17) y/o	Case, Control Experimental (Children with ADHD: *n* = 24) Comparison (control: *n* = 24)	- DSM-IV-TR - Conners Scales, Parent and Teacher form	Assessment of ADHD symptoms	HMD/ VRC	Patients with ADHD had a lower margin of safety and twice as many collisions as the controls.
Coleman et al., 2019, USA [57].	*n* = 15(6–13) y/o (12 M, 3 F)	Quasi-Experimental ADHD Children	-DSM-5 -Conners Scales, Parent and Teacher form	Concentration	HMD/ VR_CPT	Working memory training using VR led to substantial improvements in sustained attention.
Díaz-Orueta et al., 2014, Spain [58].	*n* = 57 (6B16)y/o (42 M, 15 F), ADHD children: *n* = 57	Cross-sectional Children with ADHD	-DSM-IV-TR	Assessment of ADHD symptoms	HMD/ VRC_CPT	VR_CPT is better than standard CPT in differentiating children with and without pharmacological treatment.
Eom et al. 2019, Korea [59].	*n* = 38(6–17) y/o (33 M,5 F)	Case, Control Experimental (Children with ADHD: *n* = 20) Comparison (Control: *n* = 18)	-DSM-IV	Assessment of ADHD symptoms	HMD/ VRC_CPT	ADHD and control groups exhibit comparable performances on VRC_CPT in the presence of the teacher and social cues as distractors.
Fang et al. 2019, China [60].	*n* = 140 (6–18) y/o (106 M, 34 F)	Case, Control Experimental (ADHD children: *n* = 77) Comparison (Control: *n* = 63)	-DSM-5	Assessment of ADHD symptoms	computer and a high-end VR headset/VR_CPT	VRC_CPT variables’ scores correlate with standard CPT, The Conners’ Parent Rating Scale, and Child Behavior Checklist variable scores
Gutiérrez-Maldonado, et al. 2009. Spain [61].	*n* = 20(6–11) y/o (13 M, 7 F)	Case, Control Experimental (Children with ADHD: *n* = 10) Comparison (Control: *n* = 10)	Diagnosed by clinical staff	Assessment of ADHD symptoms	VRC_CPT	Patients with ADHD make more omission errors than controls in distraction and no distraction conditions during VRC_CPT.
Hong et al., 2021, Korea [62].	*n* = 40 (9–17) y/o (31 M, 9 F)	Case, Control Experimental (Children with ADHD: *n* = 21) Comparison (Control: *n* = 19)	-DSM-5	Concentration	HMD/ VRC	VR is an effective tool to improve the concentration of children with ADHD.
Kim et al. 2020. Korea [46].	*n* = 40(8–10) y/o (35 M, 5 F)	Case, Control Experimental (game group with ADHD: *n* = 20) Comparison (non-game group with ADHD: *n* = 20)	-DSM-5	Concentration	(MR)HMD/ MR-Game	MR-Game practice reduced omission errors on the standard CPT.
Mangalmurti et al., 2020, USA [63]	*n* = 85(6–12) y/o (62 M. 23 F)	Case, Control Experimental (Children with ADHD: *n* = 45)Comparison (Control: *n* = 40)	-DSM-5	Focused attention	HMD/ VRC_CPT	VRC_CPT results indicate that shifts in the field of vision explain the link between hyperactive-impulsive symptoms and deficits in focused attention.
Muhlberger et al., 2020, Germany [64].	*n* = 128 (4–18) y/o (90 M, 38 F)	Case, Control Experimental (medicated Children with ADHD: *n* = 26, children with unmediated ADHD: *n* = 68) Comparison (Control: *n* = 34)	-DSM-IV	Assessment of ADHD symptoms	HMD/ VRC_CPT	VRC_CPT is sensitive for the detection of ADHD symptoms and medication effects.
Negut et al., 2016, Romania [65].	*n* = 75 (7–13)y/o (45 M,30 F)	Case, Control Experimental (Children with ADHD: *n* = 33) Comparison (Control: *n* = 42)	-DSM-IV-TR	Diagnosis of ADHD	HMD/ VRC_CPT	For children with ADHD, there was no significant difference between VRC_CPT and standard CPT on errors of commission, omission, and total correct responses.
OU, et al. 2020, Taiwan [66].	*n* = 3 (8–12) y/o (1 M, 2 F)	Case Study Children with ADHD	-Not identified	Concentration	VR headgear/ VR-game	Playing VR-Game improves concentration.
Parsons et al., 2007, USA [67].	*n* = 20(8–12) y/o (20 M)	Case, Control Experimental (Children with ADHD: *n* = 10) Comparison (Control: *n* = 10)	-SWAN Behavior Checklist - The Conners Rating Scale	Assessment of ADHD symptoms	HMD/ VRC_CPT	VRC_CPT measures correlate with standard CPT (Conners’ CPT) measures and parent behavior rating scale.
Pollak et al. 2010. Israel [68].	*n* = 27 (11–17) y/o (16 M, 11 F)	Case, Control Experimental (placebo children with ADHD: *n* = 19, placebo children with ADHD: *n* = 7)	-DSM-IV	Assessment of medication efficacy	HMD/ VRC_CPT	MPH reduced omission errors to a greater extent on the VR-CPT compared to the standard CPT (TOVA)
Pollak et al.,2009, Israel [69].	37 (9–17) y/o (37 M)	Case, Control Experimental (Children with ADHD: *n* = 20 Comparison (Control: *n* = 17)	-DSM-IV	Assessment of ADHD symptoms	HMD/ VRC_CPT	VRC_CPT had better sensitivity (79% vs. 65%) and equal specificity (both 94%) compared to standard CPT (TOVA) in detecting attention deficits.
Rodríguez et al. 2018, Spain [70].	*n* = 338(6–16) y/o (241 M,97 F)	Case, Control Experimental (Children with ADHD: *n* = 237 Comparison (Control: *n* = 101)	-DSM-5	Diagnosis of ADHD	HMD/ VRC_CPT	VRC_CPT is slightly better than standard CPT (TOVA) in correctly classifying the control group (66% vs 60%.) and classifying the ADHD combined group (57% vs, 50%).
Shema-Shiratzky et al., 2019, Israel [71].	*n* = 14 (8–12) y/o (11 M, 3 F)	Quasi-Experimental Children with ADHD	-DSM-5	Concentration	VR simulation is projected on a screen, treadmill/ VR training	VR-based training did not improve attention.
Tabrizi et al. 2020, Iran [72].	*n* = 48 (7–2) y/o (32 M, 16 F)	Case, Control Experimental (Children with ADHD: *n* = 16, medicated ADHD: *n* = 16 Comparison (Control: *n* = 16)	-diagnosis by psychiatrists	Memory Therapy	360° Samsung VR camera to build it. The software was stored in the VR Box camera/ VR software.	Both VR therapy and medication improved memory function in students with ADHD compared to the control group.
Tosto et al., 2020, Ireland [73].	*n* = 117 (8–9) y/o (94 M, 23 F) pilot study	Case, Control Experimental (Children with ADHD in WWL-AR, children with ADHD in WWL-AR) Comparison (Control: without any access to WWL program)	-Not identified	Education	AR-webpage to handle the display/ web-based AR	Training using AR and no AR has similar results in improving the spelling and reading skills of children with ADHD.
Yeh et al. 2020, China [74].	*n* = 68 (6–12) y/o (42 M, 26 F)	Case, Control Experimental (Children with ADHD: *n* = 37Comparison (Control: *n* = 31) (simple comparison)	-DSM-5	Diagnosis of ADHD	VR HMD, VR controller/ VRC_CPT	Machine learning models incorporating VRC_CPT and behavior rating scale data had a mean cross-validation l classification accuracy of 83%.
Zulueta et al., 2018, Spain [75].	*n* = 407 (6–16) y/o (272 M, 135 F)	Case, Control Experimental (Children with ADHD: *n* = 213 Comparison (control: *n* = 194)	-DSM-IV - The Conners Rating Scale parents’ form -interviews with children and their parents	Diagnosis of ADHD	VR/Movement sensor placed in the 3D glasses/ VRC_CPT	VRC_CPT is an effective tool to assess ADHD symptoms with a specificity of 75%, and sensitivity of 68% in diagnosing ADHD.

*ADHD* attention deficit hyperactivity disorder, *M* male, *F* female, *MA* mean age, *DSM* Diagnostic and Statistical Manual of Mental Disorders, *VR* virtual reality, *CPT* continuous performance test, *VR* virtual reality classroom, *AR* augmented reality, *MR* mixed reality, *HMD* head-mounted display

Twenty-two studies (73%) were case-controls, three (10%) were cross-sectional, two (7%) were case studies, two (7%) were quasi-experimental, and one (3%) was a randomized controlled trial (Table 2).

As for the method of ADHD diagnosis, patients had been diagnosed based on the diagnostic criteria of different versions of the DSM (DSM5 and DSM-IV) in most of the studies (*n* = 21; 70%). Three studies (10%) did not mention the method of ADHD diagnosis. In the remaining studies, inclusion in the ADHD group was based on interviews with parents and children, parental confirmation, or psychiatrists’ and clinical centers’ diagnoses (Table 2).

With respect to hardware, most of the studies utilized a head-mounted display (HMD) (*n* = 19; 63%), while others used different 3D glasses, computers, headsets, motion sensors, and robots (Table 2). In terms of the applied technologies, from the 30 studies (*n* = 27; 90%) utilizing VR, two studies used AR (*n* = 2; 7%) and only one study employed MR (*n* = 1; 3%). Notably, these studies used a VR classroom continuous performance test (VR-CPT) (*n* = 21; 70.0%), a VR classroom (VRC) environment (*n* = 3; 10%), games (*n* = 2; 7%), web-based VR (*n* = 3; 10%), and an AR robot (*n* = 1; 3%) (Table 2).

**Fig. 4:**
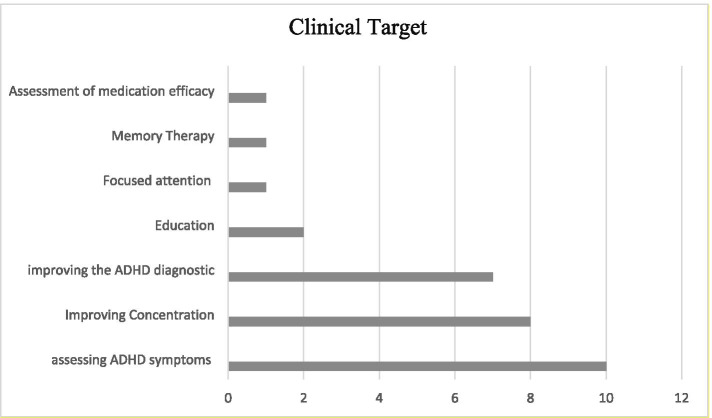
Clinical Target

The clinical objective of 10 studies (33%) was to evaluate the utility of these technologies in assessing ADHD symptoms, while seven studies (23%) focused on improving the ADHD diagnostic process (Fig. 4). In the current review, we found several studies that employed various types of VR, AR, and MR technologies, such as VR-CPT, VRC, web-based AR, and MR games, for the management of ADHD symptoms (*n* = 12; 40.0%). These studies included improving concentration (*n* = 8; 27%) by cognitive therapy [44, 62, 76], eye contact training games [46], memory training [57], and controlling a robot’s movements [45], These studies also focused on the use of VR technologies to improve academic achievement [54, 77], and in improving reading and spelling skills of children with ADHD [73]. In addition, some researchers attempted to assess the effects of medication (*n* = 2; 6%) [68, 72] and memory capacity (*n* = 1; 3.%) using these technologies [72] (Fig. 4).

### Significant findings

Most of the reviewed studies (*n* = 28; 93%) reported that using VR/AR/MR tools helped meet at least one of the clinical objectives of the study. Six of the seven studies (86%) regarding the efficacy of VR/AR/MR technologies in diagnosing ADHD found these technologies to be helpful. Similarly, eight of the nine studies (83%) assessing the ability of these technologies to manage ADHD symptoms found they were beneficial. Using VRC technology, Blume [54] found no difference between proximal and distant seat location on learning outcomes. Tosto [73] reported that training with and without AR had the same outcomes in terms of improving the spelling and reading skills of children with ADHD.

#### Quality of evidence

Additional file 1 contains the critical appraisal of the reviewed studies. Overall, most of the studies (*n* = 28; 93%) had a fair quality, and only two studies (7%) implied low/moderate quality. On the other hand, most of the studies (*n* = 24; 80%), especially case-control and cross-sectional studies, had limitations such as not evaluating the confounding factors and strategies to manage these factors.

## Discussion

Recently, VR, AR, and MR technologies have become increasingly accessible to medical researchers seeking to improve diagnostic, therapeutic, and preventive measures for patients with mental disorders [78, 79]. In particular, there has been a growing interest in the use of these technologies for the diagnosis and management of children with ADHD. We conducted a systematic search of the literature focusing on this topic.

According to our findings, VR-, AR-, and MR-based tools can be developed to improve the diagnosis and treatment of children with ADHD. Most of the reviewed studies (90%) revolved around the use of VR. Since MR technology is still in the developmental stages, most of the studies concerning its use are generally descriptive and published in lower-ranking academic journals as opposed to the studies concerning VR and AR [80]. Overall, VR appears to be a more promising technology than AR and MR for clinical purposes. On the other hand, AR and MR integrate virtual and real-world components that might prove helpful in the assessment and management of ADHD and, therefore, further investigations are warranted.

In total, 21 of the reviewed articles (70.0%) focused on the use of VR-CPT, with the first study published in 2007. In contrast, the studies regarding the application of AR and MR for this population have mostly been published in 2020. VR has been incorporated in some neuropsychological tests. Notably, VR-CPT is the most widely used non-traditional tool to diagnose and manage the symptoms of ADHD [52, 69]. In particular, this technology offers better ecological validity since patients’ sustained attention is evaluated in more realistic settings [52, 68].

Given the subjectivity of ADHD interviews and ADHD behavior rating scales, additional and more objective assessment of childhood ADHD has been recommended [81]. Unfortunately, multiple studies concerning the improvement of the ADHD diagnostic process in children have found inconsistent results when it comes to employing both objective and subjective measures [82–84]. It is, however, encouraging that according to some studies, compared to traditional CPT instrumentation, VR-CPT has superior efficiency and greater validity for ADHD diagnosis in children [58, 67, 85].

Several studies have concluded that VR-CPT technology is more efficient than traditional CPT in the treatment of children with ADHD [45, 55, 57]. This is partly because VR-CPT evokes more enjoyment in children [69]. VR-CPT technology could reportedly improve concentration for a longer period by training the patient to pay less attention to distractions [49]. Behavior therapy, which encompasses cognitive therapy and social skills training, is also reportedly effective in the rehabilitation of children with ADHD [42]. VR-enhanced behavior therapy might further ameliorate ADHD behavioral symptoms, while also enhancing treatment adherence and motivation in the patients [71]. Several studies have indicated that VR, AR, and MR technologies could incorporate effective instructional strategies to help children with ADHD learn to better manage their symptoms [86, 87]. VR-, AR-, and MR-based applications could also help these patients learn daily life skills and other helpful behaviors, while also improving their concentration and memory [88].

VR-based treatments may also have additional advantages over traditional interventions [89]. For instance, they offer a safe environment for patients and therapists and allow therapists to follow up and evaluate patients’ behavioral changes. Based on the objectives of an intervention, it is possible to modify VR-based treatments for single- or multiple-user applications. In addition, VR-based treatment options could be self-directed or carried out under a therapist’s supervision. Further research in this regard should examine the success of VR, AR, and MR technologies in the rehabilitation therapies conducted at the homes of children with ADHD as well as in clinical settings.

Based on the findings, cost-efficient HMDs are the most frequently used hardware with VR, AR, and MR technologies [90]. Compared to the traditional visualization technology, HMD has more potentials to improve the attention, general behavior, and learning ability of children with ADHD [91, 92].

Besides the merits of the reviewed studies, some of them mentioned limitations as well. First of all, the sample size of patients with ADHD in studies must be expanded to draw more accurate conclusions about the effectiveness of these technologies [50, 51, 58]. Furthermore, studies suggested that it is better to recruit two groups in such interventions to compare and acquire more reliable results [52]. Moreover, the nature of the main treatment (e.g., the time of receiving the medication and its dosage) should be taken into account since they may alter the outcome of intervention [49, 62]. Therefore, future investigations can address these limitations and, thus, improve the quality of research.

### Limitations of the study

As the search strategy was mainly focused on the titles and abstracts of relevant articles, some relevant studies may have been missed. In addition, it was not possible to access the full text of six articles, and they were excluded from the study. Potentially relevant non-English articles were excluded as well.

## Conclusion

According to the results of the reviewed studies, VR and AR technologies could be used as effective assessment tools to better assess ADHD symptoms and to improve the diagnosis of ADHD in children. Ample evidence also suggests that VR technology could augment traditional treatment options, thereby promoting their effectiveness in the management of ADHD symptoms.

## Supplementary Information

**Additional file 1.**

## Data Availability

The datasets used and/or analyzed during the current study available from the corresponding author on reasonable request.
