# Supplementary material for: The usefulness of virtual, augmented, and mixed reality technologies in the diagnosis and treatment of attention deficit hyperactivity disorder in children: an overview of relevant studies
Source: BMC Psychiatry. 2022 Jan 4;22:4. doi: 10.1186/s12888-021-03632-1 (PMC8728980; doi:10.1186/s12888-021-03632-1)
Supplement: Supplementary file 1 — Additional file 1. [file 12888_2021_3632_MOESM1_ESM.docx]

**Additional file 1: The Quality Assessment of included Studies by Joanna Briggs assessment Tools**

Case/Control studies

| References | Q_1_ | Q_2_ | Q_3_ | Q_4_ | Q_5_ | Q_6_ | Q_7_ | Q_8_ | Q_9_ | Q_10_ | Score | Quality |
| --- | --- | --- | --- | --- | --- | --- | --- | --- | --- | --- | --- | --- |
| Zulueta et al, 2018, Spain[1]. | No | Yes | Yes | Yes | Yes | No | No | Yes | Yes | Yes | 7 | Fair |
| Adams et al. 2009, USA [2]. | No | Yes | yes | Yes | Yes | No | No | Yes | Yes | Yes | 7 | Fair |
| Areces et al, 2018, Spain [3]. | No | Yes | Yes | Yes | Yes | No | No | Yes | Yes | Yes | 7 | Fair |
| Areces et al, 2016, Spain[4]. | No | Yes | Yes | Yes | Yes | No | No | Yes | Yes | Yes | 7 | Fair |
| Bioulac et al, 2012, France[5]. | No | Yes | Yes | Yes | Yes | No | No | Yes | Yes | Yes | 7 | Fair |
| Clancy et al, 2016 New Zealand. | No | Yes | Yes | Yes | Yes | Yes | Yes | Yes | Yes | Yes | 9 | Fair |
| Eom et al. 2019, Korea [6]. | No | Yes | Yes | Yes | Yes | No | No | Yes | Yes | Yes | 7 | Fair |
| Fang et al. 2019, China [7]. | No | Yes | Yes | Yes | Yes | No | No | Yes | Yes | Yes | 7 | Fair |
| Hong et al, 2021, Korea [8]. | No | Yes | Yes | Yes | Yes | No | No | Yes | Yes | Yes | 7 | Fair |
| Gutiérrez-Maldonado, et al. 2009. Spain [9]. | No | Yes | Yes | Yes | Yes | No | No | Yes | Yes | Yes | 7 | Fair |
| Kim et al. 2020. Korea [10]. | No | Unclear  Yes | Yes | Yes | Yes | No | No | Yes | Yes | Yes | 6 | Fair |
| Mangalmurti et al, 2020, USA[11] | No | Unclear | Yes | Yes | Yes | No | No | Yes | Yes | Yes | 6 | Fair |
| Muhlberger et al, 2020, Germany[12]. | No | Yes | Yes | Yes | Yes | No | No | Yes | Yes | Yes | 7 | Fair |
| Negut et al, 2016, Romania[13]. | No | Yes | Yes | Yes | Yes | No | No | Yes | Yes | Yes | 7 | Fair |
| Parsons et al, 2007, USA[14]. | No | Yes | Yes | Yes | Yes | No | No | Yes | Yes | Yes | 7 | Fair |
| Pollak et al. 2010. Israel[15]. | No | Unclear | Yes | Yes | Yes | No | No | Yes | Yes | Yes | 6 | Fair |
| Pollak et al,2009, Israel [16]. | No | Yes | Yes | Yes | Yes | No | No | yes | Yes | Yes | 7 | Fair |
| Rodríguez et al. 2018, Spain[17]. | No | Yes | Yes | Yes | Yes | No | No | Yes | Yes | Yes | 7 | Fair |
| Tabrizi et al. 2020, Iran[18]. | No | Yes | Yes | Yes | Yes | No | No | Yes | Yes | Yes | 7 | Fair |
| Tosto et al, 2020, Ireland[19]. | No | Yes | Not Applicable | Unclear | Yes | No | No | yes | Yes | Yes | 5 | L/M |
| Yeh et al. 2020, China[20]. | No | Yes | Yes | Yes | Yes | No | No | Yes | Yes | Yes | 7 | Fair |
| Cho et al, 2002, China [21]. | No | Unclear | Unclear | Yes | Yes | No | No | Yes | Yes | Yes | 5 | L/M |

Questions for Case/Control studies

| 1. were the groups comparable other than the presence of disease in cases or the absence of disease in controls? |
| --- |
| 1. Were cases and controls matched appropriately? |
| 1. Were the same criteria used for identification of cases and controls? |
| 1. Was exposure measured in a standard, valid and reliable way? |
| 1. Was exposure measured in the same way for cases and controls? |
| 1. Were confounding factors identified? |
| 1. Were strategies to deal with confounding factors stated? |
| 1. Were outcomes assessed in a standard, valid and reliable way for cases and controls? |
| 1. Was the exposure period of interest long enough to be meaningful? |
| 1. Was appropriate statistical analysis used? |

Randomize Control Trial Study:

| References | Q_1_ | Q_2_ | Q_3_ | Q_4_ | Q_5_ | Q_6_ | Q_7_ | Q_8_ | Q_9_ | Q_10_ | Q_11_ | Q_12_ | Score | Quality |
| --- | --- | --- | --- | --- | --- | --- | --- | --- | --- | --- | --- | --- | --- | --- |
| Bioulac et al. 2018, France[22]. | Yes | Yes | Yes | No | No | Yes | Yes | Yes | Yes | Yes | Yes | Yes | 10 | Fair |

| 1. Was true randomization used for assignment of participants to treatment groups? |
| --- |
| 1. Was allocation to treatment groups concealed? |
| 1. Were treatment groups similar at the baseline? |
| 1. Were participants blind to treatment assignment? |
| 1. Were those delivering treatment blind to treatment assignment? |
| 1. Were outcomes assessors blind to treatment assignment? |
| 1. Were treatment groups treated identically other than the intervention of interest? |
| 1. Was follow up complete and if not, were differences between groups in terms of their follow up adequately described and analyzed? |
| 1. Were participants analyzed in the groups to which they were randomized? |
| 1. Were outcomes measured in the same way for treatment groups? |
| 1. Were outcomes measured in a reliable way? |
| 1. Was appropriate statistical analysis used? |

Cross Sectional Studies

| References | Q_1_ | Q_2_ | Q_3_ | Q_4_ | Q_5_ | Q_6_ | Q_7_ | Q_8_ | Score | Quality |
| --- | --- | --- | --- | --- | --- | --- | --- | --- | --- | --- |
| Blume et al. 2018, Germany.[23] | Yes | Yes | Yes | Yes | No | No | Yes | Yes | 6 | Fair |
| Areces et al, 2020, Spain[24]. | Yes | Yes | Yes | Yes | No | No | Yes | Yes | 6 | Fair |
| Díaz-Orueta et al, 2014, Spain[25]. | Yes | Yes | Yes | Yes | No | No | Yes | Yes | 6 | Fair |

| 1. Were the criteria for inclusion in the sample clearly defined? |
| --- |
| 1. Were the study subjects and the setting described in detail? |
| 1. Was the exposure measured in a valid and reliable way? |
| 1. Were objective, standard criteria used for measurement of the condition? |
| 1. Were confounding factors identified? |
| 1. Were strategies to deal with confounding factors stated? |
| 1. Were the outcomes measured in a valid and reliable way? |
| 1. Was appropriate statistical analysis used?   Case studies |

| References | Q_1_ | Q_2_ | Q_3_ | Q_4_ | Q_5_ | Q_6_ | Q_7_ | Q_8_ | Score | Quality |
| --- | --- | --- | --- | --- | --- | --- | --- | --- | --- | --- |
| Arpaia et, al. 2020, Italy[26]. | Yes | Yes | Yes | No | Yes | Yes | No | No | 5 | Fair |
| OU, et al. 2020, Taiwan[27]. | Yes | yes | Yes | No | Yes | Yes | No | Unclear  no | 5 | Fair |

| 1. Were patient’s demographic characteristics clearly described? |
| --- |
| 1. Was the patient’s history clearly described and presented as a timeline? |
| 1. Was the current clinical condition of the patient on presentation clearly described? |
| 1. Were diagnostic tests or assessment methods and the results clearly described? |
| 1. Was the intervention(s) or treatment procedure(s) clearly described? |
| 1. Was the post-intervention clinical condition clearly described? |
| 1. Were adverse events (harms) or unanticipated events identified and described? |
| 1. Does the case report provide takeaway lessons? |
| 1. Were patient’s demographic characteristics clearly described? |

Quasi Experimental studies

| References | Q_1_ | Q_2_ | Q_3_ | Q_4_ | Q_5_ | Q_6_ | Q_7_ | Q_8_ | Q_9_ | Score | Quality |
| --- | --- | --- | --- | --- | --- | --- | --- | --- | --- | --- | --- |
| Shema-Shiratzky et al, 2019, Israel[28]. | Yes | No | Yes | No | Yes | Yes | No | No | Yes | 7 | Fair |
| Coleman et al, 2019, USA[29]. | yes | No | No | No | Yes | Yes | No | Yes | Yes | 7 | Fair |

| 1. Is it clear in the study what is the ‘cause’ and what is the ‘effect’ (i.e. there is no confusion about which variable comes first)? |
| --- |
| 1. Were the participants included in any comparisons similar? |
| 1. Were the participants included in any comparisons receiving similar treatment/care, other than the exposure or intervention of interest? |
| 1. Was there a control group? |
| 1. Were there multiple measurements of the outcome both pre and post the intervention/exposure? |
| 1. Was follow up complete and if not, were differences between groups in terms of their follow up adequately described and analyzed? |
| 1. Were the outcomes of participants included in any comparisons measured in the same way? |
| 1. Were outcomes measured in a reliable way? |
| 1. Was appropriate statistical analysis used? |

**References:**

1. Zulueta A, Díaz-Orueta U, Crespo-Eguilaz N, Torrano F: **Virtual reality-based assessment and rating scales in ADHD diagnosis**. *Psicologia Educativa* 2019, **25**(1):13-22.

2. Adams R, Finn P, Moes E, Flannery K, Rizzo A: **Distractibility in attention/deficit/hyperactivity disorder (ADHD): The virtual reality classroom**. *Child Neuropsychology* 2009, **15**(2):120-135.

3. Areces D, Dockrell J, Garcia T, Gonzalez-Castro P, Rodriguez C: **Analysis of cognitive and attentional profiles in children with and without ADHD using an innovative virtual reality tool**. *Plos One* 2018, **13**(8).

4. Areces D, Rodriguez C, Garcia T, Cueli M, Gonzalez-Castro P: **Efficacy of a Continuous Performance Test Based on Virtual Reality in the Diagnosis of ADHD and Its Clinical Presentations**. *Journal of Attention Disorders* 2018, **22**(11):1081-1091.

5. Bioulac S, Lallemand S, Rizzo A, Philip P, Fabrigoule C, Bouvard MP: **Impact of time on task on ADHD patient's performances in a virtual classroom**. *European Journal of Paediatric Neurology* 2012, **16**(5):514-521.

6. Eom H, Kim KK, Lee S, Hong YJ, Heo J, Kim JJ, Kim E: **Development of Virtual Reality Continuous Performance Test Utilizing Social Cues for Children and Adolescents with Attention-Deficit/Hyperactivity Disorder**. *Cyberpsychology, Behavior, and Social Networking* 2019, **22**(3):198-204.

7. Fang Y, Han D, Luo H: **A virtual reality application for assessment for attention deficit hyperactivity disorder in school-aged children**. *Neuropsychiatric Disease and Treatment* 2019, **15**:1517-1523.

8. Hong N, Kim JJ, Kwon JH, Eom H, Kim E: **Effect of Distractors on Sustained Attention and Hyperactivity in Youth With Attention Deficit Hyperactivity Disorder Using a Mobile Virtual Reality School Program**. *Journal of Attention Disorders* 2021.

9. Gutiérrez-Maldonado J, Letosa-Porta A, Rus-Calafell M, Penaloza-Salazar C: **The assessment of attention deficit hyperactivity disorder in children using continous performance tasks in virtual environments**. *Anuario de Psicologia* 2009, **40**(2):211-222.

10. Kim S, Ryu J, Choi Y, Kang Y, Li H, Kim K: **Eye-contact game using mixed reality for the treatment of children with attention deficit hyperactivity disorder**. *IEEE Access* 2020, **8**:45996-46006.

11. Mangalmurti A, Kistler WD, Quarrie B, Sharp W, Persky S, Shaw P: **Using virtual reality to define the mechanisms linking symptoms with cognitive deficits in attention deficit hyperactivity disorder**. *Scientific Reports* 2020, **10**(1).

12. Mühlberger A, Jekel K, Probst T, Schecklmann M, Conzelmann A, Andreatta M, Rizzo AA, Pauli P, Romanos M: **The Influence of Methylphenidate on Hyperactivity and Attention Deficits in Children With ADHD: A Virtual Classroom Test**. *Journal of Attention Disorders* 2020, **24**(2):277-289.

13. Neguț A, Jurma AM, David D: **Virtual-reality-based attention assessment of ADHD: ClinicaVR: Classroom-CPT versus a traditional continuous performance test**. *Child Neuropsychology* 2017, **23**(6):692-712.

14. Parsons TD, Bowerly T, Buckwalter JG, Rizzo AA: **A controlled clinical comparison of attention performance in children with ADHD in a virtual reality classroom compared to standard neuropsychological methods**. *Child Neuropsychology* 2007, **13**(4):363-381.

15. Pollak Y, Shomaly HB, Weiss PL, Rizzo AA, Gross-Tsur V: **Methylphenidate effect in children with ADHD can be measured by an ecologically valid continuous performance test embedded in virtual reality**. *CNS Spectr* 2010, **15**(2):125-130.

16. Pollak Y, Weiss PL, Rizzo AA, Weizer M, Shriki L, Shalev RS, Gross-Tsur V: **The utility of a continuous performance test embedded in virtual reality in measuring ADHD-related deficits**. *Journal of developmental and behavioral pediatrics : JDBP* 2009, **30**(1):2-6.

17. Rodríguez C, Areces D, García T, Cueli M, González-Castro P: **Comparison between two continuous performance tests for identifying ADHD: Traditional vs. virtual reality**. *International Journal of Clinical and Health Psychology* 2018, **18**(3):254-263.

18. Tabrizi M, Manshaee G, Ghamarani A, Rasti J: **Comparison of the effectiveness of virtual reality with medication on the memory of attention deficit hyperactivity disorder students**. *International Archives of Health Sciences* 2020, **7**(1):37-42.

19. Tosto C, Hasegawa T, Chiazzese G, Treacy R, Merlo G, Chifari A, Mangina E: **"AHA - ADHD AUGMENTED" - PARTICIPANTS' CHARACTERISTICS**. In: *Edulearn19: 11th International Conference on Education and New Learning Technologies.* edn. Edited by Chova LG, Martinez AL, Torres IC; 2019: 5637-5645.

20. Yeh SC, Lin SY, Wu EHK, Zhang KF, Xiu X, Rizzo A, Chung CR: **A Virtual-Reality System Integrated with Neuro-Behavior Sensing for Attention-Deficit/Hyperactivity Disorder Intelligent Assessment**. *IEEE Transactions on Neural Systems and Rehabilitation Engineering* 2020, **28**(9):1899-1907.

21. Cho BH, Ku J, Jang D, Lee J, Oh M, Kim H, Lee J, Kim J, Kim I, Kim S: **Clinical test for Attention Enhancement System**. *Studies in health technology and informatics* 2002, **85**:89-95.

22. Bioulac S, Micoulaud-Franchi JA, Maire J, Bouvard MP, Rizzo AA, Sagaspe P, Philip P: **Virtual Remediation Versus Methylphenidate to Improve Distractibility in Children With ADHD: A Controlled Randomized Clinical Trial Study**. *Journal of Attention Disorders* 2020, **24**(2):326-335.

23. Blume F, Göllner R, Moeller K, Dresler T, Ehlis AC, Gawrilow C: **Do students learn better when seated close to the teacher? A virtual classroom study considering individual levels of inattention and hyperactivity-impulsivity**. *Learning and Instruction* 2019, **61**:138-147.

24. Areces D, Rodríguez C, García T, Cueli M: **Is an ADHD observation-scale based on DSM criteria able to predict performance in a virtual reality continuous performance test?** *Applied Sciences (Switzerland)* 2020, **10**(7).

25. Díaz-Orueta U, Garcia-López C, Crespo-Eguílaz N, Sánchez-Carpintero R, Climent G, Narbona J: **AULA virtual reality test as an attention measure: Convergent validity with Conners Continuous Performance Test**. *Child Neuropsychology* 2014, **20**(3):328-342.

26. Arpaia P, Duraccio L, Moccaldi N, Rossi S: **Wearable Brain-Computer Interface Instrumentation for Robot-Based Rehabilitation by Augmented Reality**. *IEEE Transactions on Instrumentation and Measurement* 2020, **69**(9):6362-6371.

27. Ou YK, Wang YL, Chang HC, Yen SY, Zheng YH, Lee BO: **Development of virtual reality rehabilitation games for children with attention-deficit hyperactivity disorder**. *Journal of Ambient Intelligence and Humanized Computing* 2020, **11**(11):5713-5720.

28. Shema-Shiratzky S, Brozgol M, Cornejo-Thumm P, Geva-Dayan K, Rotstein M, Leitner Y, Hausdorff JM, Mirelman A: **Virtual reality training to enhance behavior and cognitive function among children with attention-deficit/hyperactivity disorder: brief report**. *Developmental neurorehabilitation* 2019, **22**(6):431-436.

29. Coleman B, Marion S, Rizzo A, Turnbull J, Nolty A: **Virtual Reality Assessment of Classroom - Related Attention: An Ecologically Relevant Approach to Evaluating the Effectiveness of Working Memory Training**. *Frontiers in Psychology* 2019, **10**.
